# ID 166 - Evolução das Demandas de Tecnologias em Saúde no SUS: análise das avaliações da Conitec (2012-2024)

**DOI:** 10.5327/2237-9622.2025.v34s1.166

**Published:** 2025-11-25

**Authors:** Aíla Coelho do Carmo, Denis Satoshi Komoda, Nayara Castelano Brito

**Keywords:** Avaliação de Tecnologias em Saúde, ATS, Conitec, acesso, equidade, Política Nacional de Gestão de Tecnologias em Saúde, Sistema Único de Saúde, SUS

## Abstract

**Introdução:**

A partir da publicação da Lei n.º 12.401, em 28 de abril de 2011, alterando a Lei n.º 8.080, de 19 de setembro de 1990, a assistência terapêutica e a incorporação de tecnologias em saúde no âmbito do Sistema Único de Saúde (SUS) passaram a ser estabelecidas no Ministério da Saúde sob assessoria da Comissão Nacional de Incorporação de Tecnologias em Saúde (Conitec). O processo de Avaliação de Tecnologias em Saúde (ATS) – parte da Política Nacional da Gestão de Tecnologias em Saúde de 2010 – favorece a sustentabilidade do sistema, transparência e qualidade das tecnologias, além de garantir os princípios constitucionais do SUS, em especial equidade e integralidade. Nesse contexto, os dados do painel "Conitec em Números" entre 2012 e 2024 mostram que as tecnologias em saúde submetidas à Conitec perpassam um total de 1.224 demandas avaliadas, entre as quais 74,1% representam medicamentos, 15,8% procedimentos e 10,1% produtos. O aprimoramento das informações contidas neste painel de acompanhamento permite identificar aspectos importantes quanto ao alcance desta política e populações beneficiadas.

Os objetivos deste trabalho serão categorizar as demandas submetidas a Conitec nos temas de saúde atingidos pós-recomendações e realizar uma análise temporal no período entre 2012 e 2024.

**Método:**

Foi realizada uma pesquisa documental no site atual da Conitec para extrair dados das demandas submetidas à Comissão. Os dados de interesse foram: "Motivo da solicitação", "Nome da Tecnologia", "Tema da Saúde" e "Demandante". Esses dados foram apresentados por meio de frequências e utilizados para classificação das demandas nos temas da saúde. Além disso, foi apresentada uma análise de tendência temporal.

**Resultados:**

Entre 2012 e 2024 foram avaliados 819 medicamentos, 74 produtos e 168 procedimentos. As demandas que tiveram como motivo da solicitação a incorporação representaram 85% (906) do total, seguido por 8,3% para exclusão e 6% para ampliação de uso. As demais demandas tiveram como motivo de avaliação: restrição de uso, alteração e reavaliação. A análise temporal não mostra tendência de crescimento tanto no número de avaliações gerais quanto por cada uma das categorias apresentadas anteriormente. Os seis temas em saúde mais frequentes foram infectologia (14,5%), oncologia (11%), hematologia (7,2%), neurologia (6,7%), pneumologia (5,7%), genética (5,7%) e cardiovascular (5%).

**Conclusão:**

Os dados sobre a ampla gama de especialidades envolvidas mostram a capacidade do SUS em responder de maneira integrada e coordenada às necessidades de saúde da população contribuindo para a garantia da integralidade e equidade, com eficácia e sustentabilidade das políticas de saúde. Pode-se observar o papel da Conitec na incorporação de tecnologias duras, relacionada à incorporação de técnicas mais modernas e menos invasivas, potencializando resultados clínicos e uma melhor experiência do paciente. A predominância das demandas por incorporação seguido pelas exclusões indica que os avanços tecnológicos e o incremento de robustez nas evidências científicas pressionam a inclusão de novas tecnologias no sistema de saúde. O acompanhamento dos temas em saúde no qual estas tecnologias se inserem permite conhecer as especialidades atendidas, refletindo a dinâmica das demandas com a priorização equilibrando inovação com sustentabilidade do sistema.

